# Development of Fluorescence-Tagged SARS-CoV-2 Virus-like Particles by a Tri-Cistronic Vector Expression System for Investigating the Cellular Entry of SARS-CoV-2

**DOI:** 10.3390/v14122825

**Published:** 2022-12-19

**Authors:** Young-Sheng Chang, Li-Wei Chu, Zan-Yu Chen, Joh-Sin Wu, Wen-Chi Su, Chia-Jui Yang, Yueh-Hsin Ping, Cheng-Wen Lin

**Affiliations:** 1 Department of Medical Laboratory Science and Biotechnology, China Medical University, Taichung 404394, Taiwan; 2 Graduate Institute of Biomedical Sciences, China Medical University, Taichung 404394, Taiwan; 3 Department and Institute of Pharmacology, National Yang Ming Chiao Tung University, Taipei 11221, Taiwan; 4 PhD Program for Health Science and Industry, China Medical University, Taichung 404394, Taiwan; 5 International Master’s Program of Biomedical Sciences, China Medical University, Taichung 404394, Taiwan; 6 Department of Internal Medicine, Far Eastern Memorial Hospital, New Taipei City 22060, Taiwan; 7 Institute of Biophotonics, National Yang Ming Chiao Tung University, Taipei 11221, Taiwan; 8 Department of Medical Laboratory Science and Biotechnology, Asia University, Wufeng, Taichung 413305, Taiwan

**Keywords:** SARS-CoV-2, virus-like particle, fluorescence labeling, cell entry, ACE2, fusion, endocytosis, rab5

## Abstract

Severe acute respiratory syndrome-related coronavirus-2 (SARS-CoV-2) has caused the pandemic that began late December 2019. The co-expression of SARS-CoV-2 structural proteins in cells could assemble into several types of virus-like particles (VLPs) without a viral RNA genome. VLPs containing S proteins with the structural and functional properties of authentic virions are safe materials to exploit for virus-cell entry and vaccine development. In this study, to generate SARS-CoV-2 VLPs (SCoV2-SEM VLPs) composed of three structural proteins including spike (S), envelop (E) protein and membrane (M) protein, a tri-cistronic vector expression system was established in a cell line co-expressing SARS-CoV-2 S, E and M proteins. The SCoV2-SEM VLPs were harvested from the cultured medium, and three structure proteins were confirmed by Western blot assay. A negative-stain TEM assay demonstrated the size of the SCoV2-SEM VLPs with a diameter of about 90 nm. To further characterize the infectious properties of SCoV2-SEM VLPs, the VLPs (atto647N-SCoV2-SEM VLPs) were fluorescence-labeled by conjugation with atto-647N and visualized under confocal microscopy at a single-particle resolution. The results of the infection assay revealed that atto647N-SCoV2-SEM VLPs attached to the surface of the HEK293T cells at the pre-binding phase in a ACE2-dependent manner. At the post-infection phase, atto647N-SCoV2-SEM VLPs either fused with the cellular membrane or internalized into the cytoplasm with mCherry-rab5-positive early endosomes. Moreover, fusion with the cellular membrane and the internalization with early endosomes could be inhibited by the treatment of camostat (a pharmacological inhibitor of TMPRSS2) and chlorpromazine (an endocytosis inhibitor), respectively. These results elucidated that SCoV2-SEM VLPs behave similarly to the authentic live SARS-CoV-2 virus, suggesting that the development of SCoV2-SEM VLPs provide a realistic and safe experimental model for studying the infectious mechanism of SARS-CoV-2.

## 1. Introduction

Severe acute respiratory syndrome-related coronavirus-2 (SARS-CoV-2) causes coronavirus disease 2019 (COVID-19) with asymptomatic or mild to severe symptoms such as acute respiratory distress, pneumonia, and eventually death. Over 647 million COVID-9 cases, including over 6.6 million confirmed deaths, have been reported as of middle December 2022 [1]. SARS-CoV-2 is a novel betacoronavirus containing a single-stranded positive sense RNA genome of nearly 30 kb, with about 80% and 50% nucleotide sequence identity with SARS-CoV and Middle East respiratory syndrome coronavirus (MERS-CoV), respectively [2]. The SARS-CoV-2 genome comprises functional open reading frames (ORFs) that encode a large polyprotein replicase (ORF1a and ORF1ab), spike (S), envelope (E), membrane (M) and nucleocapsid (N), as well as some putative ORFs coding for accessory proteins [2,3]. SARS-CoV-2 replicase is cleaved into 16 non-structural proteins (NSPs) by viral papain-like protease in NSP3 and 3C-like protease (main protease, NSP5) [3,4]. Mature replicase proteins form a replication/transcription complex (RTC) to initiate RNA-dependent RNA polymerase (RdRp), the NSP12-mediated synthesis of negative- and positive-strand viral genome RNAs and subgenomic mRNAs [5]. SARS-CoV-2 proteases, RdRp and other replicase proteins are responsible for viral transcription and replication, and are viral direct targets for developing antiviral agents against SARS-CoV-2. 

Of the four essential structural proteins, S protein is the main protein for mediating virus entry and determining cell tropism. SARS-CoV-2 S proteins containing 1273 amino acids share approximately 77% amino acid sequence similarities with SARS-CoV, showing a unique genomic feature with a furin cleavage site motif of Pro-Arg-Arg-Ala (PRRA) located at the junction of the S1 and S2 subunits [6]. The receptor-binding domain (RBD) within the S1 subunit of SARS-CoV-2 S protein is responsible for binding to human angiotensin-converting enzyme 2 (hACE2) as the key receptor for virus entry [7]. SARS-CoV-2 infects specific types of human bronchiolar and submucosal gland epithelial cells in the respiratory tract, as well as alveolar macrophages and type II pneumocytes in the lung, due to the significant expression of ACE2 and transmembrane serine protease 2 (TMPRSS2) [8]. Among the other structural proteins, E and M proteins are required for efficient SARS-CoV-2 assembly with the integration of S trimers on the virion surface to form the crown-like appearance [9,10]. The N protein forms helical ribonucleoprotein complexes with viral RNA genomes, interacting with M proteins during the budding of virions [11]. 

SARS-CoV-2 structural proteins could assemble into several types of virus-like particles (VLPs) in mammalian cells, including S-E-M-N, S-E-M, E-M-N, and E-M, without a viral RNA genome [12,13,14,15,16]. VLPs containing S protein are recognized as valuable biological materials for the study of virus-cell entry and to exploit for vaccine design. Most reports focus on the expression systems to produce SARS-CoV-2 S-based VLPs and to study the vaccine application of viral VLPs, due to their safety and ease in production [12,14]. In addition to vaccine development, SARS-CoV-2 S-based VLPs exhibit unique biological properties in the research of virus-cell entry. Although S protein-based pseudo-viruses in adenoviral or lentiviral systems have been commonly used to examine the entry events of SARS-CoV-2 [17,18], SARS-CoV-2 S-based VLPs with the other two or three structural proteins show molecular, structural and morphological properties of authentic virions, providing the function of the structural proteins involved in the cell entry during the processes of the viral life cycle.

This study produced and characterized SARS-CoV-2 VLPs in a mammalian expression system with the transfection of a tri-cistronic vector containing Wuhan-1 (WH1)-strain S, E and M genes. The study also examined the biological properties of Atto647N-labeled VLPs in the cell entry pathway of ACE2-mediated endocytosis. Image analysis demonstrated the critical steps during cell entry of SARS-CoV-2 SEM VLPs into HEK293T-ACE2 cells in comparison to HEK293T mock cells. This study provided new insights into the production of VLPs in tri-cistronic vector expression system, and the studying and visualization of the cell entry of the SARS-CoV-2 replication cycle.

## 2. Materials and Methods

### 2.1. Cells

Human embryonic kidney HEK-293T cells were cultured in Dulbecco’s Modified Eagle Medium (DMEM) (Thermo Fisher Scientific, Waltham, MA, USA) complemented with 10% fetal bovine serum (FBS) (Thermo Fisher Scientific, Waltham, MA, USA), 100 U/mL penicillin-streptomycin (PS) (HyClone, Logan, UT, USA) and 1 μg/mL Amphotericin B (HyClone™), and incubated under 5% CO_2_ at 37 °C. Human lung epithelial Calu-3 cells utilized for testing in vitro viral characteristics were grown in Minimal Essential Medium (MEM) (HyClone, Logan, UT, USA) complemented with 10% FBS, 100 U/mL PS, 1 μg/mL Amphotericin B, 100 mM sodium pyruvate (Thermo Fisher Scientific, Waltham, MA, USA) and 0.1% nonessential amino acids 100X (Corning, NY, USA) at 37 °C.

### 2.2. Construction of Recombinant Plasmids Containing S, E, and M Genes of SARS-CoV-2 Reference Strain Wuhan-Hu-1 (WH1)

**Figure 1:**
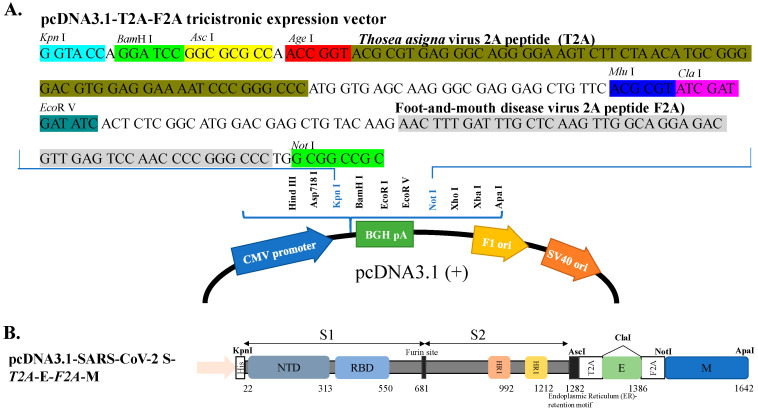
The construction of SARS-CoV2 S-E-M tri-expressing plasmid. (**A**) The sequence of pcDNA3.1-T2A-F2A tri-cistronic vector depicting the linker [BamHI-AscI-AgaI-T2A-MluI-ClaI-EcoRV-F2A] was inserted into KpnI/NotI sites to form a tri-cistronic expression backbone containing T2A and F2A self-cleaving peptides. (**B**) The construction of pcDNA3.1-SARS-CoV-2 S-T2A-E-F2A-M depicted that the cDNA of spike inserted into KpnI/ AscI sites, E inserted into ClaI and M inserted into NotI/ApaI sites.

For the co-expression of S, E and M proteins in the transfected cells, the S, E and M genes of the SARS-CoV-2 Wuhan-Hu-1 (WH1) strain were cloned into a tri-cistronic expression vector. In this study, the mammalian expression vector pcDNA3.1-HisC was modified as a triple-cistronic expression vector and inserted with the linker [BamHI-AscI-AgaI-T2A-MluI-ClaI-EcoRV-F2A] into the KpnI/NotI sites (Figure 1A). The linker contains multiple cloning sites and two 2A self-cleaving peptides F2A from the foot-and-mouth disease virus and T2A from the *Thosea asigna* virus. The modified pcDNA3.1-HisC used in this study was named pcDNA3.1-*T2A*-*F2A* tri-cistronic vector, of which the restriction enzyme map and nucleotide sequence were shown in Figure 1A. The codon-optimized synthetic membrane (M) protein gene of the SARS-CoV-2 WH1 strain in the plasmid pET-32a(+)-M protein (Supplemental Figure S1A,B) was kindly provided by Dr. Shih-Wein Li (Institute Pasteur of Shanghai, Chinese Academy of Sciences, Shanghai, China). The M gene was amplified using PCR with the specific primer pair listed in Supplemental Figure S1C, and then cloned into the restriction enzyme sites NotI/ApaI of pcDNA3.1-*T2A*-*F2A*, and the resultant plasmid was named pcDNA3.1-*T2A*-*F2A*-nCoV M. Next, the envelope (E) gene of the SARS-CoV-2 WH1 strain (Supplemental Figure S2A,B) in the plasmid pCAG.2-HA-SARS-CoV-2-E-HA (kindly provided by Professor Che Alex Ma, Academia Sinica, Taiwan) was amplified by PCR with the specific primer pair listed in Supplemental Figure S2C, and then cloned into the restriction enzyme site *Cla*I of pcDNA3.1-*T2A*-*F2A*-CoV-2 M. The subsequent recombinant plasmid was called resultant pcDNA3.1-*T2A*-CoV-2 E-*F2A*-CoV-2 M. Finally, the codon-optimized synthetic spike (S) protein gene of the SARS-CoV-2 WH-1 strain in the plasmid pCMV3-2019-nCoV-Spike (S1+S2)-long (Supplemental Figure S3A,B), kindly provided by Professor Che Alex Ma, was amplified using PCR with the specific primer pair listed in Supplemental Figure S3C, and then constructed into the restriction enzyme sites KpnI/AscI of pcDNA3.1-*T2A*-CoV-2 E-*F2A*-CoV-2 M, of which the recombinant plasmid was called pcDNA3.1-SARS-CoV-2 WH1 S-*T2A*-E-*F2A*-M (Figure 1B). The sequences of the SARS-CoV-2 WH-1 S, E, and M genes in recombinant vector pcDNA3.1-SARS-CoV-2 WH1 S-*T2A*-E-*F2A*-M were verified by restriction enzyme analysis and Sanger sequencing with specific primers. 

### 2.3. Stable Transfected Cell Co-Expressing SARS-CoV-2 WH1 S, E, and M Proteins

To establish stable transfected cell lines co-expressing to produce S, E, and M proteins, HEK293T cells were grown to 70% confluence in a 6-wells plate, and then transfected with 2 µg of pcDNA3.1-SARS-CoV-2 WH1 S-*T2A*-E-*F2A*-M using jetPRIME (Polyplus, Dandenong, South VIC, AU) according to the manufacturer’s guidelines. The transfected cells were selected by treatment with 1000 µg/mL G418 48 h post transfection. After a 3-week treatment with G418, the expression of S, E, and M in the stable cell lines transfected were further analyzed using real-time RT-PCR, immunofluorescent staining and Western blotting assays.

### 2.4. Real-Time RT-PCR Assays

To measure the mRNA co-expression of SARS-CoV-2 WH1 S, E, and M genes in transfected cells, the total RNAs were extracted from the stable transfected cells with pcDNA3.1-SARS-CoV-2 WH1 S-*T2A*-E-*F2A*-M using the PureLink Micro-to-Midi Total RNA Purification System kit (Invitrogen, Carlsbad, CA, USA) and synthesis cDNA with SuperScript™ III Reverse Transcriptase (Invitrogen, Carlsbad, CA, USA), and then evaluated using SYBR Green-based real-time PCR assay with SARS-CoV-2 WH1 S, E, and M-specific primer pairs. The S-specific primer pair had forward primer 5′-GCAATGAAGCCCAGCCAGATGTA-3′and reverse primer 5′-GTGGCTAAGAACCTGAATGAG-3′. The E-specific primers were forward primer 5′-ACAGGTACGTTAATAGTTAATAGCGT-3′ and reverse primer 5′-ATATTGCAGCAGTACGCACACA-3′. The M-specific primer pair contained forward primer 5′-TTTGTGCTTGCTGCTGTTTAC-3′ and reverse primer 5′-GAGTGGCACGTTGAGAAGAAT-3′. In addition, the glyceraldehyde 3-phosphate dehydrogenase (GAPDH)-specific primers pairs were forward primer 5′-TGCACCACCAACTGCTTAG-3′ and reverse primer 5′-GATGCAGGGATGATGTTC-3′. Finally, relative mRNA expression levels of SARS-CoV-2 WH1 S, E, and M genes in transfected cells were normalized by the GAPDH expression.

### 2.5. Immunofluorescent Assay

The cells were fixed with 4% paraformaldehyde for 15 min, washed with phosphate buffered saline (PBS) twice, and permeabilized with 0.1% Triton X-100 in PBS for 15 min at room temperature. After being blocked in 3% BSA for 1 h, the cells were incubated with primary antibodies, including mouse anti-CoV-2 spike and rabbit anti-SARS-CoV-2 membrane (M) antibodies (Genetex, Hsinchu, Taiwan), overnight at 4 °C, and washed with PBS twice; they then reacted in the dark with fluorophore-conjugated secondary antibodies, such as goat anti-rabbit IgG- DyLight594 and goat anti-mouse IgG- DyLight594 (Genetex, Hsinchu, Taiwan), respectively. After staining with DAPI for 15 min, the cells were observed and recorded by fluorescent microscopy (Olympus, BX50, Tokyo, Japan).

### 2.6. Western Blotting Assay

The cells were lysed by Thermo Scientific RIPA Lysis and Extraction Buffer (Thermo Fisher Scientific, Waltham, MA, USA). The cultured media were concentrated by being added into the dialysis tubing cellulose membrane with a molecular weight cut-off (MWCO) of 14,000 Da (Sigma-Aldrich, St. Louis, MO, USA), placed onto a bed of polyethylene glycol-8000 (PEG-8000, Sigma-Aldrich, St. Louis, MO, USA), and covered with more PEG-8000. After quantifying the concentration by bicinchoninic acid (BCA) assay (Thermo Fisher Scientific, Waltham, MA, USA), the lysate and concentrated cultured media (30 μg per sample) were mixed with a 2X SDS-PAGE (sodium dodecyl sulfate–polyacrylamide gel electrophoresis) sample buffer, denatured at 95 °C for 5 min, separated by 8% to 15% SDS-PAGE gels, and then transferred to nitrocellulose membranes. Resultant blots were blocked with 5% skim milk in TBST (Tris Buffered Saline with Tween 20) for 1 h at room temperature, then reacted with appropriately diluted primary antibodies (including mouse anti-beta-actin (Novous, Centennial, CO, USA) at 1:5000, mouse anti-CoV-2 spike (Genetex, Hsinchu, Taiwan) at 1:5000, rabbit anti-CoV-2 envelope (Genetex, Hsinchu, Taiwan) at 1:3000, and rabbit anti-CoV-2 membrane (Genetex, Hsinchu, Taiwan) at 1:5000 in TBST, respectively) at 4 °C overnight. After washing with TBST twice, the blots were further incubated with goat-anti rabbit and goat-anti mouse secondary antibodies conjugated to horseradish peroxidase (HRP) at 1:5000 in TBST for 1 h at room temperature, washed with TBST twice and then reacted with Trident plus Western HRP Substrate (Genetex, Hsinchu, Taiwan). The chemiluminescence signal of immuno-reactive bands in Western blots was measured by Multi-function Gel Image System (MultiGel-21) (Gentaur, San Jose, CA, USA).

### 2.7. Analysis of Virus Like Particles Using Western Blotting and Transmission Electron Microscopy (TEM)-Negative Staining

To examine the formation of virus like particles (VLPs) released from the stable cell lines co-expressing SARS-CoV-2 WH1 S, E and M proteins, the cultured media of the cell lines were harvested, clarified of cell debris by centrifugation at 1000 rpm for 3 min, and then concentrated in the dialysis tubes with 14,000 Da-MWCO by PEG-8000 at 4 °C. The protein subunits of SARS-CoV-2 WH1 VLPs in concentrated cultured media were analyzed by Western blot assay with specific antibodies against S, E and M proteins, as mentioned above. Moreover, 10 μL of concentrated cultured media were seeded on TEM grid support films of carbon 400 mesh (PELCO^®^, 01754-F, Fresno, CA, USA) for 1 min, dried by filter papers, and subsequently stained with 0.1% phosphotungstic acid (Sigma-Aldrich, St. Louis, MO, USA) for 50 sec. After the grids were air-dried in the dark overnight, the particle size and morphology of VLPs were measured and photographed by a 70 kV transmission electron microscope (HITACHI HT7700, Tokyo, Japan).

### 2.8. Condensing and Fluorescence Labelling of Virus Like Particles 

VLP-containing media, which were collected from VLP-producing HEK293T cells, were centrifugated at 9000× *g* for 30 min to remove cell debris. VLPs were pelleted from a VLP-containing supernatant by ultra-centrifugation at 47,000 rpm in a Beckman 50.2Ti rotor for 3.5 h. VLP pellets were resuspended in a HNE buffer (5 mM HEPES, 150 mM NaCl, and 0.1 mM EDTA, pH 7.4) and further concentrated by 100 kDa cut-off ultrafiltration spin columns (GE Healthcare, Chicago, IL, USA). Concentrated VLPs were labeled with Atto647N-NHS ester (Sigma-Aldrich, St. Louis, MO, USA), with maximum absorption at 646 nm and maximum emission at 664 nm. Briefly, VLP solutions of 100 μL were mixed with 2 nmol of Atto647N NHS ester in an HNE buffer for 45 min at room temperature. The unconjugated dye was separated from the Atto647N-labeled VLPs by a Sephadex G-25 column (GE Healthcare, Chicago, IL, USA). The fractions containing Atto647N-labeling VLPs were detected by a multimode microplate reader (TECAN 200/200Pro, Männedorf, Switzerland) and stored at −80 °C.

### 2.9. Infection Assay for SARS-CoV-2 VLPs

HEK293T or HEK293T-ACE2 cells were first seeded in 35 mm imaging dishes with a No. 1.5 polymer coverslip bottom (ibidi) and transfected with mCherry-Rab5 for 24 h prior to VLP infection. To initiate VLP infection, Atto647N-VLPs were incubated with the cells at 4 °C for 30 min as pre-binding, and then the VLP-bound cells were transferred to 37 °C. At indicated time points, VLP-infected cells were fixed by 4% *v*/*v* paraformaldehyde at room temperature for 15 min for staining. Cellular membrane was stained with WGA488. The confocal images were acquired using the Olympus FV1000 confocal system equipped with a 100x Oil DIC objective lens with an N.A. of 1.4. 

### 2.10. Statistical Analysis

The data of the three independent experiments were represented as mean ± standard deviation (S.D.) and then evaluated by Schee’s post-hoc test of the one-way ANOVA. A *p* value less than 0.05 was considered to have a statistical significance. 

## 3. Results

### 3.1. Establishment of a Sable Transfected Cell Line Co-Expressing SARS-CoV-2 S, E and M Proteins

**Figure 2:**
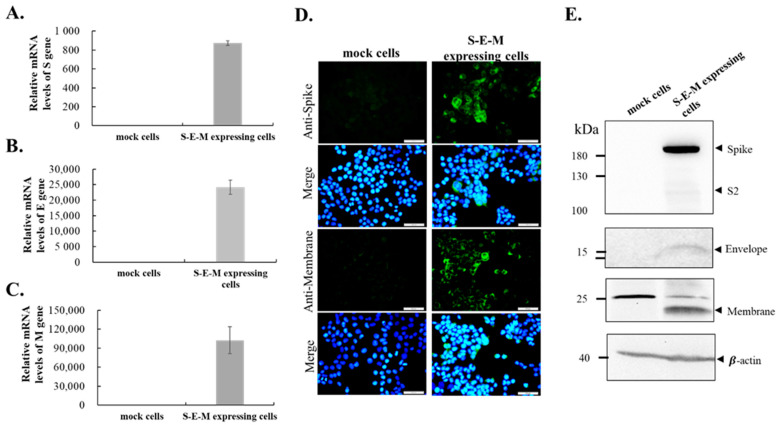
The expression of SARS-CoV2 spike, envelope and membrane protein in S-E-M expressing cells. The real time RT-PCR showed the transcription of spike, envelope, and membrane gene in S-E-M expressing cells (**A**–**C**). (**D**) The immuno-fluorescence imaging illustrated the spike and membrane protein accumulated in S-E-M expressing cells. (**E**) The Western blotting showed the protein expression of spike, envelope, and membrane in S-E-M expression cells. The mock cells only had a minor non-species background.

**Figure 3:**
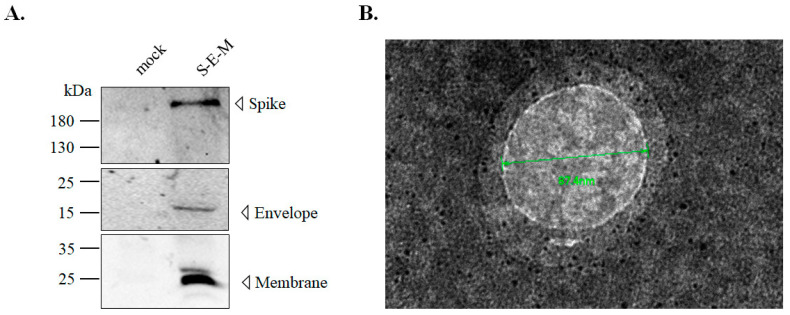
Isolation of SCoV2-SEM VLPs from the culture medium of S-E-M-expressing cells. (**A**) Western blotting ascertained the presence of recombinant S, E, and M proteins in the culture media of S-E-M-expressing cells, but not mock cells. (**B**) The negative-stain TEM image illustrated that the size of SCoV2-SEM VLPs was around 87 nm.

To create a cell line co-expressing SARS-CoV-2 S, E and M proteins, the S, E, and M genes of the SARS-CoV-2 WH1 strain were cloned into the cloning sites KpnI/AscI, ClaI, and NotI/ApaI of the T2A and F2A-linked tri-cistronic expression vector pcDNA3.1-*T2A*-*F2A*, respectively (Figure 1). The resultant recombinant plasmid pcDNA3.1-SARS-CoV-2 WH1 S-*T2A*-E-*F2A*-M was subsequently transfected into HEK-293T cells to generate the stable cell lines after treatment with G418 for three weeks. Next, the co-expression of SARS-CoV-2 WH1 S, E and M genes in stable transfected cells was further examined using real-time RT-PCR, immunofluorescent staining, and Western blotting assays (Figure 2A–E). Intracellular mRNA and protein expressions for each individual gene were detected in stable transfected cells but not in mock cells, by real-time RT-PCR and immunofluorescent staining (Figure 2A–D). Moreover, Western blotting analysis indicated that S (182 kDa), E (15 kDa) and M (23 kDa) recombinant proteins were determined in the cell lysate of stable transfected cells, but not mock cells (Figure 1E). Thus, the stable transfected cell line co-expressing SARS-CoV-2 WH1 S, E and M was successfully established. Then, the formation and release of SARS-CoV-2 VLPs from S, E and M co-expressing cells were explored in the cultured media (Figure 3). Western blotting ascertained the presence of recombinant S, E, and M proteins in the cultured media S, E and M co-expressing cells, but not in the mock cells (Figure 3A). In addition, negative-stain TEM assay demonstrated the image of SARS-CoV-2 VLPs on a TEM mesh grid, showing a diameter of about 90 nm (Figure 3B). Therefore, the result indicated the production and release of SARS-CoV-2 VLPs from stable transfected cells, with the T2A and F2A-linked tri-cistronic expression vector containing SARS-CoV-2 WH1 strain S, E and M genes. 

### 3.2. Generation of Fluorescence-Labeled SARS-CoV-2 VLPs

**Figure 4:**
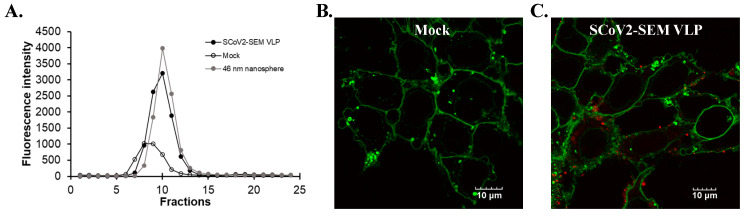
The generation of the Atto647N-tagged SCoV2-SEM VLPs. (**A**) Fluorescence intensity profiles of elution from a Sephadex G-25 size-exclusion column were shown. The signals of Atto647N-tagged SCoV2-SEM VLPs were indicated as black circles. Background signals were detected in SCoV2-SEM VLP-free medium (white circles). The signals of 46 nm nanospheres were shown in gray circles. (**B**,**C**) The confocal microscopy images of HEK293T-ACE2 cells incubated with atto647N fractions from SCoV2-SEM VLP-free medium (**B**) or medium with Atto647N-tagged SCoV2-SEM VLPs. (**C**) Fluorescent images of SCoV2-SEM VLPs were indicated in red. Scale bar, 10 μm.

To directly visualize the cellular entry of SARS-CoV-2 VLPs, the VLPs were first labeled with a fluorescence dye (atto647N) that can be linked with the E protein of the SARS-CoV-2 VLPs by conjugating atto647N-NHS ester with the amino group of the E protein. Accordingly, the atto647N-labeling did not affect the infectivity in several viruses, such as Dengue virus (DENV) and Zika virus (ZIKV) [19,20,21]. Atto647N-labeled SARS-CoV-2 VLPs (atto-647N-SARS-CoV-2 VLPs) were separated from free atto647N by a Sephadex G-25 size-exclusion column. Compared to the fractions of the 46 nm fluorosphere signals (Figure 4A, gray circles), the fluorescent signals of atto647N were detected from fraction 8 to fraction 12 (Figure 4A, black circles), indicating that the size of atto-647N-SARS-CoV-2 VLPs are larger than 46 nm fluorospheres. Although the VLP-free medium from the 293T-ACE2 cell culture displayed atto647N signals from fraction 7 to fraction 11 (Figure 4A, white circles), there were no detectable signals on the cell surface in the binding assay (Figure 4B), suggesting that those signals might be noise. In contrast, atto647N-SARS-CoV-2 VLPs could be visualized on the surface of 293T-ACE2 cells at single-particle resolution (Figure 4C).

### 3.3. Infection of SARS-CoV-2 VLPs

**Figure 5:**
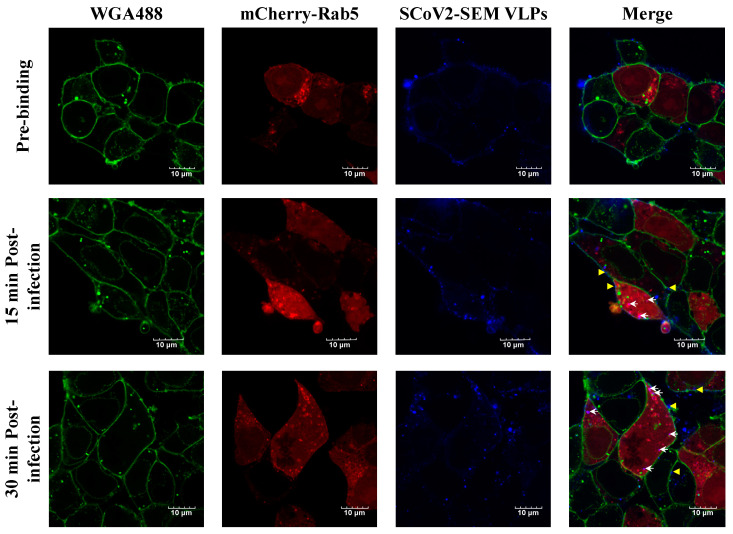
The visualization of Atto647N-tagged SCoV2-SEM VLP infection in HEK293T-ACE2 cells. HEK293T-ACE2 cells were incubated with Atto647N-tagged SCoV2-SEM VLPs at 4 °C for 30 min, considered as viral pre-binding, and the temperature was switched to 37 °C, considered as viral infection, for time indicated. Cellular membrane stained with WGA488 was shown in green, mCherry-Rab5-indicated early endosome was shown in red, and Atto647N-tagged SCoV2-SEM VLPs were depicted in blue. White arrows indicated that SCoV2-SEM VLPs were co-localized with early endosomes. Yellow arrows indicated that SCoV2-SEM VLPs were fused with cell membrane. Scale bar, 10 μm.

Given that SARS-CoV-2 infection can be through the endocytic pathway [7,22], we visualized SARS-CoV-2 VLP infection by using a confocal microscopy. Confocal imaging revealed that atto647N-SARS-CoV-2 VLPs were attached on the surface of 293T-ACE2 cells at the pre-binding phase (Figure 5, top panel). At 15 and 30-min post-infection, the signals of atto647N were detected in the cellular membrane, suggesting the occurrence of membrane fusion between atto647N-SARS-CoV-2 VLPs and the cellular membrane (Figure 5, middle and bottom panels, yellow arrowhead). Moreover, the signals of atto647N were also co-localized with mCherry-Rab5 in the cytoplasm after infection (Figure 5, middle and bottom panels, white arrow), suggesting the co-localization of the SARS-CoV-2 VLPs and early endosomes. These results implied that the cell entry of SARS-CoV-2 VLPs is similar to the entry of the authentic live SARS-CoV-2 virus. 

### 3.4. ACE2-Dependent Infection of SARS-CoV-2 VLPs 

**Figure 6:**
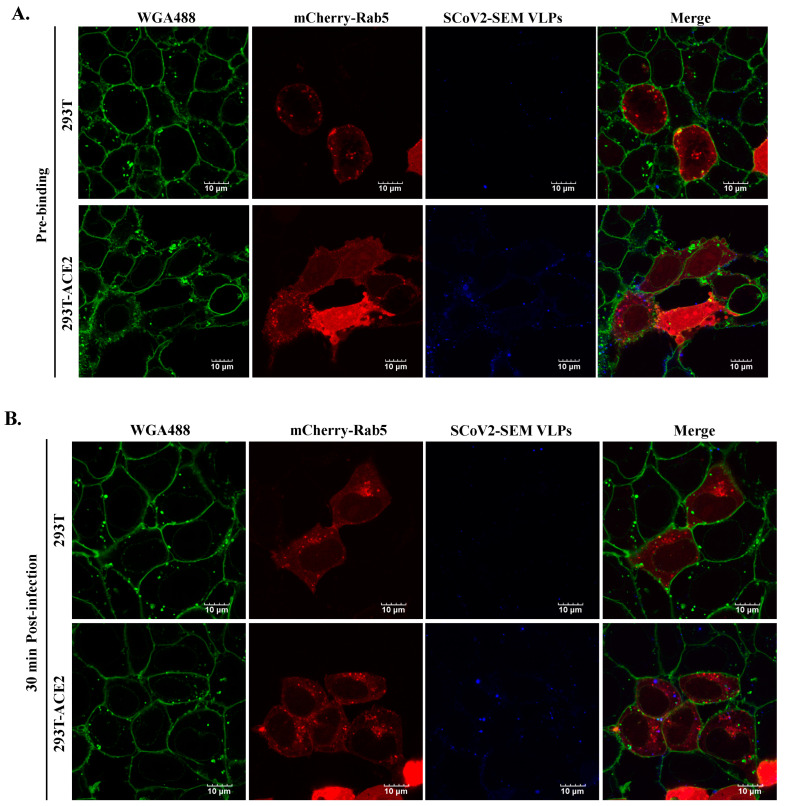
The ACE2-dependent infection of SCoV2-SEM VLPs. Atto647N-tagged SCoV2-SEM VLPs infected either HEK-293T or HEK-293T-ACE2 according to the procedure described previously. The confocal images were acquired after 4 °C incubation for 30 min (**A**). The post-infection images were acquired at 30 min after temperature switch (**B**). Cellular membrane stained with WGA488 was shown in green, mCherry-Rab5-indicated early endosome was shown in red, and Atto647N-tagged SCoV2-SEM VLPs were depicted in blue. Scale bar, 10 μm.

ACE-2 is required for the infection of SARS-CoV-2 [9,10,23]. To validate whether the infection of SARS-CoV-2 VLPs is dependent on ACE2, HEK293T cells were infected by atto647N-SARS-CoV-2 VLPs in the presence or absence of ACE2 expression. As shown in Figure 6A, compared to ACE-2-expressing cells (bottom panel), the binding of SARS-CoV-2 VLPs on the cell surface at the pre-binding phase was barely detected in the absence of ACE2 expression (top panel). Furthermore, neither membrane fusion nor co-localization with early endosomes was observed when 293T cells without ACE2 expression were infected with atto647N-SARS-CoV-2 VLPs (Figure 6B). These observations confirmed that the infection of SARS-CoV-2 VLPs is ACE2-dependent.

### 3.5. The Infection of SARS-CoV-2 VLPs Was Affected by Various Inhibitors

**Figure 7:**
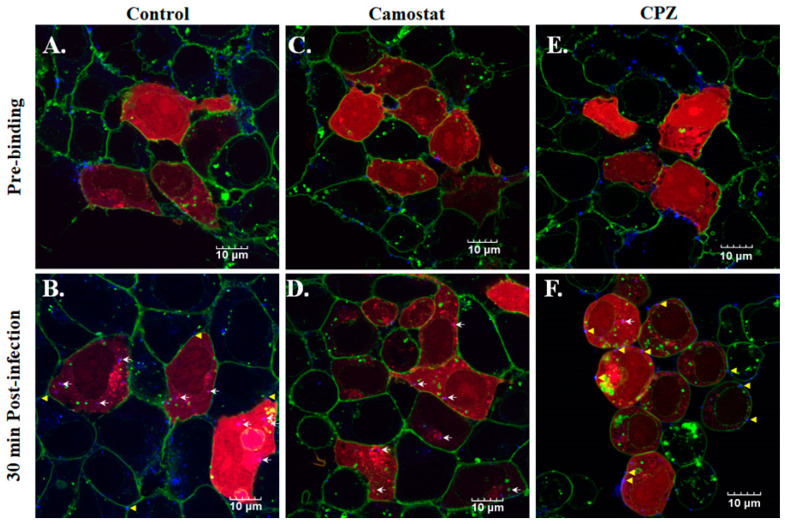
The effects of various inhibitors on SCoV2-SEM VLPs infection. Atto647N-tagged SCoV2-SEM VLPs infected HEK-293T-ACE2 (**A**,**B**), in the presence of either 100 μM of Camostat, a pharmacological inhibitor of TMPRSS2 (**C**,**D**), or 50 μM of CPZ, a clathrin-mediated endocytosis inhibitor (**E**,**F**). Cellular membrane stained with WGA488 was shown in green, mCherry-Rab5-indicated early endosome was shown in red, and Atto647N-tagged SCoV2-SEM VLPs were depicted in blue. The confocal merging images were acquired at the pre-binding step (**A**,**C**,**E**) and at 30 min post-infection (**B**,**D**,**F**). White arrows indicated the co-localization of SCoV2-SEM VLPs with early endosomes. Yellow arrows indicated the membrane fusion of SCoV2-SEM VLPs. Scale bar, 10 μm.

SARS-CoV-2 infection is initiated by the binding of the SARS-CoV-2 spike protein with ACE2, followed by the cleavage of the spike protein mediated by TMPRSS2 [23]. To investigate the infection of SARS-CoV-2 VLPs requiring the protease activity of TMPRSS2, 293T-ACE2 cells were infected by atto647N-SARS-CoV-2 VLPs in a treatment of 100 mM of Camostat, a pharmacological inhibitor of TMPRSS2. Compared to the control group, Camostat did not affect the binding of SARS-CoV-2 VLPs with 293T-ACE2 cells, but reduced the events of membrane fusion and cellular internalization (Figure 7A–D). To further confirm that SARS-CoV-2 VLP infection can occur through the endocytic pathway, 293T-ACE2 cells were infected by atto647N-SARS-CoV-2 VLPs in the presence of 50 mM of chlorpromazine (CPZ), an endocytosis inhibitor. Confocal imaging results depicted that most of the fluorescent SARS-CoV-2 VLPs remained on the surface of the 293T-ACE2 cells (Figure 7E,F), suggesting that CPZ treatment inhibited the cellular internalization of SARS-CoV-2 VLPs but not the binding of SARS-CoV-2 VLPs with 293T-ACE2 cells.

## 4. Discussion

This study demonstrated the production of SARS-CoV-2 VLPs in the cultured media of SARS-CoV-2 S, E, and M co-expressing cells (Figure 1, Figure 2, Figure 3 and Figure 4). The study discovered a suitable and manipulatable reverse genetic system, including the construction of a tri-cistronic expression vector for the concurrent and synchronized expression of SARS-CoV-2 S, E, and M in a stably transfected cell line (Figure 1 and Figure 2), and the release and formation of VLPs assembled by S, E, and M proteins (Figure 3 and Figure 4). In this system, the recombinant tri-cistronic expression vector, comprised of SARS-CoV-2 S, E, and M genes into one tri-cistronic vector, could easier and more constantly generate SARS-CoV-2 VLPs in transfected cells compared to the transient expression in those cells co-transfected with three plasmids containing an individual gene [12,13,14,15,16]. In addition, the relative expression profile of the structural proteins S, E and M, in transfected cells using this tri-cistronic vector expression system, was visualized by Western blotting assays (Figure 2 and Figure 3), presenting a similar pattern to the S, E and M expression levels in infected cells with SARS-CoV-2 VOCs [24]. 

In SARS-CoV-2 studies, the real virus or infectious materials must be manipulated in biosafety level 3 laboratories, limiting the investigation rate of the SARS-CoV-2 life cycle. The application of substitutes such as pseudovirus, viral protein-expressing cells and VLPs have been developed for SARS-CoV-2 studying [25]. The pseudovirus of SARS-CoV-2 was a kind of chimeric virus with SARS-CoV-2 spike and other structure proteins derived from different viruses, such as lentivirus or Vesicular stomatitis virus (VSV) [26,27]. In previous studies, the pseudovirus of SARS-CoV-2 combined various protease inhibitor treatments to demonstrate that both cell membrane fusion and the endocytosis pathway would exist in the SARS-CoV-2 entry [23]. A pseudovirus system, containing the SARS-CoV-2 spike protein and the Firefly luciferase reporter gene, was used to investigate the ability of traditional medicine ingredients to inhibit viral entry [28,29]. On the other hand, SARS-CoV-2 spike-expressing cells were used to mimic the interaction of spike and host facts and the cytopathic effects of SARS-CoV-2 infection [30,31,32,33]. Compared with pseudovirus and viral protein-expressing cells, VLPs self-assembled from SARS-CoV-2 spike, envelope, and membrane proteins were the most similar components of the SARS-CoV-2 virion. Indeed, the EM imaging in Figure 3B revealed that the size of the VLPs (87 nm) was similar to that of SARS-CoV-2 (~100 nm) [34].

In the current study, we exhibited a realistic experimental model of SARS-CoV-2 that is available in a BSL-2 instead of BSL-3 setting to investigate viral infection. In line with previous studies [8,9,10,23,35,36], the infection of atto647N-SARS-CoV-2 VLPs is in an ACE-2-dependent and TMPRSS2-dependent manner (Figure 6 and Figure 7). Moreover, the co-localization of atto647N-SARS-CoV-2 VLPs with mCherry-rab5 that was inhibited by the treatment of CPZ (an endocytosis inhibitor) is consistent with earlier studies (Figure 5 and Figure 7) [7,22]. Taken together, these results elucidate that VLPs behave as an authentic live SARS-CoV-2 virus.

The labeling of SARS-CoV-2 VLPs with fluorescence could be very useful to visualize either viral entry or assembly in target cells. The fusion of structural proteins of SARS-CoV-2 (including S, M and N proteins) with a fluorescence protein such as green fluorescence protein (GFP) had been used to visualize SARS-CoV-2 VLPs in cells [13,25]. Given that the molecular weight of fluorescent proteins is around 25–30 kDa, it is possible that tagging with fluorescence proteins may hinder the biological functions of SARS-CoV-2 structure proteins. Because we have used atto647N—a small fluorescent molecule with a molecular weight less than one kDa—to label DENV and ZIKV and found no effects on viral infectivity [19,20,21], using atto647N provided an alternative labeling approach to label SARS-CoV-2 VLPs in the current study. As shown in Figure 5 and Figure 6, atto647N-SARS-CoV-2 VLPs could bind specifically to 293T cells expressing ACE2 and internalize into cells through endocytosis, suggesting that atto647N labeling did not affect the infectious properties of the VLPs. 

The generation of SARS-CoV-2 VLPs provides a useful tool to benefit the elucidation of the life-cycle of SARS-CoV-2 in host cells. In addition to viral entry, SARS-CoV-2 VLPs can be used to identify host factors involved in SARS-CoV-2 assembly and egression. These VLP strategies can also be applied to the discovery of anti-SARS-CoV-2 drug targets and the developments of vaccines and drug delivery systems [37,38]. Indeed, various VLPs-based vaccines have been approved by the FDA or tested in clinical trials against various infectious diseases, such as HPV, Hepatitis B, Hepatitis E, Influenza Virus, Ebola Virus and COVID-19 [39,40,41,42,43,44,45]. Furthermore, defective interfering particles (DIPs) are the other types of virus-like particles that contain a normal set of viral structure proteins packaged with a subgenomic defective-interfering RNA, which interfere specifically with the intracellular replication of the parental virus [46]. SARS-CoV-2 VLPs can be further developed into DIPs against SARS-CoV-2 specifically. Taken together, the development of SARS-CoV-2 VLPs can provide a full range of applications, from fundamental to clinical research.

## 5. Conclusions

This study demonstrated the co-expression of SARS-CoV-2 S, E and M proteins by a tri-cistronic vector expression system in cells, leading to the assembly of these three structural proteins and the release of SCoV2 VLPs in the cultured media. Image analysis by negative-stain TEM and confocal microscopy plus fluorescence dye labeling assay indicated that the SCoV2 SEM VLPs exhibited structural and biological properties similar to authentic virions, including the size, morphology, receptor usage, membrane fusion, and endocytic entry. Treatment with the inhibitors of TMPRSS2 and clathrin-dependent endocytosis displayed the unique features of SCoV2 SEM VLPs in the cell entry pathways via ACE2/TMPRSS2 membrane fusion and ACE2-mediated endocytosis. Thus, the study provides a convenient tool for the production of SARS-CoV-2 VLPs, elucidating the single-particle resolution of SARS-CoV-2 during the early steps of viral replication, particularly at the steps of attachment, membrane fusion and endocytic entry of the virus. The study suggests that SCoV2-SEM VLPs could be a realistic and safe experimental model for further studying the infectious mechanism of emerging SARS-CoV-2 variants. 

## Data Availability

Not Applicable.

## Supplementary Materials

The following supporting information can be downloaded at: https://www.mdpi.com/article/10.3390/v14122825/s1, Figure S1: Nucleotide sequence and cloning of codon optimized synthetic gene of SARS-CoV-2 membrane (M) protein; Figure S2: Nucleotide/amino acid sequences and cloning of SARS-CoV-2 envelope (E) protein gene; Figure S3: Nucleotide/amino acid sequences and cloning of SARS-CoV-2 Wuhan-Hu-1 spike (S) protein gene.
