# Supplementary material for: Development of Fluorescence-Tagged SARS-CoV-2 Virus-like Particles by a Tri-Cistronic Vector Expression System for Investigating the Cellular Entry of SARS-CoV-2
Source: Viruses. 2022 Dec 19;14(12):2825. doi: 10.3390/v14122825 (PMC9786960; doi:10.3390/v14122825)
Supplement: Supplementary file 1 [file viruses-14-02825-s001.zip › viruses-2096551-supplementary.pdf]

A. GCGGCCGCTCATGGCGGACAGCAACGGTACCATCACCGTGGAGGAACTGAAGAAACTGCTGGAGCA
GTGGAACCTGGTTATCGGCTTCCTGTTTCTGACCTGGATTTGCCTGCTGCAATTCGCGTACGCGAACC
GTAACCGTTTTCTGTACATCATCAAGCTGATTTTCCTGTGGCTGCTGTGGCCGGTGACCCTGGCGTGC
TTTGTGCTGGCGGCGGTTTACCGTATCAACTGGATTACCGGTGGCATCGCGATTGCGATGGCGTGCC
TGGTTGGTCTGATGTGGCTGAGCTATTTTATCGCGAGCTTCCGTCTGTTTGCGCGTACCCGTAGCATG
TGGAGCTTCAACCCGGAGACCAACATCCTGCTGAACGTGCCGCTGCACGGCACCATTCTGACCCGTC
CGCTGCTGGAGAGCGAACTGGTGATCGGTGCGGTTATTCTGCGTGGCCACCTGCGTATTGCGGGTCA
CCACCTGGGCCGTTGCGACATCAAGGATCTGCCGAAAGAAATTACCGTGGCGACCAGCCGTACCCTG
AGCTACTATAAGCTGGGTGCGAGCCAGCGTGTTGCGGGTGACAGCGGTTTTGCGGCGTACAGCCGTT
ATCGTATCGGCAACTACAAACTGAACACCGACCACAGCAGCAGCAGCGATAACATTGCGCTGCTGGTT
CAGGGGCCC

B. MADSNGTITVEELKKLLEQWNLVIGFLFLTWICLLQFAYANRNRFLYIIKLIFLWLLWPVTLACFVLAAVYRIN
WITGGIAIAMACLVGLMWLSYFIASFRLFARTRSMWSFNPETNILLNVPLHGTILTRPLLESELVIGAVILRG
HLRIAGHHLGRCDIKDLPKEITVATSRTLSYYKLGASQRVAGDSGFAAYSRYRIGNYKLNTDHSSSSDNIA
LLVQ

C. nCoV-M NotI-F: 5’-ATATGCGGCCGCTCATGGCGGACAGCAACGGT-3’
nCoV-M ApaI-R: 5’-ATATGGGCCCCTGAACCAGCAGCGCAAT-3’

Supplemental FigureS1. Nucleotide sequence and cloning of codon optimized synthetic gene of SARS-CoV-2 membrane (M) protein. The codon optimized synthetic membrane (M) protein gene of SARS-CoV-2 Wuhan-Hu-1 strain (NCBI Reference Sequence: NC_045512.2) was chemically synthesized and cloned in the plasmid pET-32a(+)-M protein (kindly provided by Dr. Shih-Wein Li at Institut Pasteur of Shanghai, Chinese Academy of Sciences). Nucleotide and deduced amino acid sequences of SARS-CoV-2 M protein gene were listed (A, B), and used as the template for PCR amplification with a specific primer pair (C). Finally, the codon optimized synthetic gene of SARS-CoV-2 M protein was cloned into the restriction enzyme sites NotI/ApaI of pcDNA3.1/HisC-T2A-F2A.

A. ATCGATATGTACTCATTCGTTTCGGAAGAGACAGGTACGTTAATAGTTAATAGCGTACTTCTTTTTCTTGC
TTTCGTGGTATTCTTGCTAGTTACACTAGCCATCCTTACTGCGCTTCGATTGTGTGCGTACTGCTGCAAT
ATTGTTAACGTGAGTCTCGTAAAACCTTCTTTTTACGTTTACTCTCGTGTTAAAAATCTGAATTCTTCTAG
AGTTCCTGATCTTCTGGTCATCGAT

B. MYSFVSEETGTLIVNSVLLFLAFVVFLLVTLAILTALRLCAYCCNIVNVSLVKPSFYVYSRVKNLNSSRVPDL
LV

C. nCoV-E ClaI-F: 5’-ATATATCGATATGTACTCATTCGTTTCGG-3’
nCoV-E ClaI-R: 5’-ATATATCGATGACCAGAAGATCAGGAAC-3’

Supplemental FigureS2. Nucleotide/amino acid sequences and cloning of SARS-CoV-2 envelope (E) protein gene. The envelope (E) protein gene of SARS-CoV-2 Wuhan-Hu-1 strain (NCBI Reference Sequence: NC_045512.2) was chemically synthesized and cloned in the plasmid pCAG.2-HA-SARS-CoV-2-E-HA (kindly provided by Professor Che Alex Ma at Genomics Research Center, Academia Sinica, Taiwan). Nucleotide and deduced amino acid sequences of SARS-CoV-2 E protein gene were listed (A, B), and used as the template for PCR amplification with a specific primer pair (C). Finally, the codon optimized synthetic gene of SARS-CoV-2 E protein was cloned into the restriction enzyme site ClaI of pcDNA3.1/HisC-T2A-F2A-nCoV M.

A. GGTACCAATGTTTGTGTTCCTGGTGCTGCTGCCACTGGTGTCCAGCCAGTGTGTGAACCTGACCACCAGGACCCAACTTCCTCCTGCCTACACCAACTCCTTCACCAGGGGAGTCTACTACCCTGACAAGGTGTTCAGGTCCTCT
GTGCTGCACAGCACCCAGGACCTGTTCCTGCCATTCTTCAGCAATGTGACCTGGTTCCATGCCATCCATGTGTCTGGCACCAATGGCACCAAGAGGTTTGACAACCCTGTGCTGCCATTCAATGATGGAGTCTACTTTGCCAGCA
CAGAGAAGAGCAACATCATCAGGGGCTGGATTTTTGGCACCACCCTGGACAGCAAGACCCAGTCCCTGCTGATTGTGAACAATGCCACCAATGTGGTGATTAAGGTGTGTGAGTTCCAGTTCTGTAATGACCCATTCCTGGGAGT
CTACTACCACAAGAACAACAAGTCCTGGATGGAGTCTGAGTTCAGGGTCTACTCCTCTGCCAACAACTGTACCTTTGAATATGTGAGCCAACCATTCCTGATGGACTTGGAGGGCAAGCAGGGCAACTTCAAGAACCTGAGGGAG
TTTGTGTTCAAGAACATTGATGGCTACTTCAAGATTTACAGCAAACACACACCAATCAACCTGGTGAGGGACCTGCCACAGGGCTTCTCTGCCTTGGAACCACTGGTGGACCTGCCAATTGGCATCAACATCACCAGGTTCCAGAC
CCTGCTGGCTCTGCACAGGTCCTACCTGACACCTGGAGACTCCTCCTCTGGCTGGACAGCAGGAGCAGCAGCCTACTATGTGGGCTACCTCCAACCAAGGACCTTCCTGCTGAAATACAATGAGAATGGCACCATCACAGATGC
TGTGGACTGTGCCCTGGACCCACTGTCTGAGACCAAGTGTACCCTGAAATCCTTCACAGTGGAGAAGGGCATCTACCAGACCAGCAACTTCAGGGTCCAACCAACAGAGAGCATTGTGAGGTTTCCAAACATCACCAACCTGTGT
CCATTTGGAGAGGTGTTCAATGCCACCAGGTTTGCCTCTGTCTATGCCTGGAACAGGAAGAGGATTAGCAACTGTGTGGCTGACTACTCTGTGCTCTACAACTCTGCCTCCTTCAGCACCTTCAAGTGTTATGGAGTGAGCCCAA
CCAAACTGAATGACCTGTGTTTCACCAATGTCTATGCTGACTCCTTTGTGATTAGGGGAGATGAGGTGAGACAGATTGCCCCTGGACAAACAGGCAAGATTGCTGACTACAACTACAAACTGCCTGATGACTTCACAGGCTGTGTG
ATTGCCTGGAACAGCAACAACCTGGACAGCAAGGTGGGAGGCAACTACAACTACCTCTACAGACTGTTCAGGAAGAGCAACCTGAAACCATTTGAGAGGGACATCAGCACAGAGATTTACCAGGCTGGCAGCACACCATGTAAT
GGAGTGGAGGGCTTCAACTGTTACTTTCCACTCCAATCCTATGGCTTCCAACCAACCAATGGAGTGGGCTACCAACCATACAGGGTGGTGGTGCTGTCCTTTGAACTGCTCCATGCCCCTGCCACAGTGTGTGGACCAAAGAAGA
GCACCAACCTGGTGAAGAACAAGTGTGTGAACTTCAACTTCAATGGACTGACAGGCACAGGAGTGCTGACAGAGAGCAACAAGAAGTTCCTGCCATTCCAACAGTTTGGCAGGGACATTGCTGACACCACAGATGCTGTGAGGG
ACCCACAGACCTTGGAGATTCTGGACATCACACCATGTTCCTTTGGAGGAGTGTCTGTGATTACACCTGGCACCAACACCAGCAACCAGGTGGCTGTGCTCTACCAGGATGTGAACTGTACTGAGGTGCCTGTGGCTATCCATGC
TGACCAACTTACACCAACCTGGAGGGTCTACAGCACAGGCAGCAATGTGTTCCAGACCAGGGCTGGCTGTCTGATTGGAGCAGAGCATGTGAACAACTCCTATGAGTGTGACATCCCAATTGGAGCAGGCATCTGTGCCTCCTA
CCAGACCCAGACCAACAGCCCAAGGAGGGCAAGGTCTGTGGCAAGCCAGAGCATCATTGCCTACACAATGAGTCTGGGAGCAGAGAACTCTGTGGCTTACAGCAACAACAGCATTGCCATCCCAACCAACTTCACCATCTCTGT
GACCACAGAGATTCTGCCTGTGAGTATGACCAAGACCTCTGTGGACTGTACAATGTATATCTGTGGAGACAGCACAGAGTGTAGCAACCTGCTGCTCCAATATGGCTCCTTCTGTACCCAACTTAACAGGGCTCTGACAGGCATT
GCTGTGGAACAGGACAAGAACACCCAGGAGGTGTTTGCCCAGGTGAAGCAGATTTACAAGACACCTCCAATCAAGGACTTTGGAGGCTTCAACTTCAGCCAGATTCTGCCTGACCCAAGCAAGCCAAGCAAGAGGTCCTTCATT
GAGGACCTGCTGTTCAACAAGGTGACCCTGGCTGATGCTGGCTTCATCAAGCAATATGGAGACTGTCTGGGAGACATTGCTGCCAGGGACCTGATTTGTGCCCAGAAGTTCAATGGACTGACAGTGCTGCCTCCACTGCTGACA
GATGAGATGATTGCCCAATACACCTCTGCCCTGCTGGCTGGCACCATCACCTCTGGCTGGACCTTTGGAGCAGGAGCAGCCCTCCAAATCCCATTTGCTATGCAGATGGCTTACAGGTTCAATGGCATTGGAGTGACCCAGAATG
TGCTCTATGAGAACCAGAAACTGATTGCCAACCAGTTCAACTCTGCCATTGGCAAGATTCAGGACTCCCTGTCCAGCACAGCCTCTGCCCTGGGCAAACTCCAAGATGTGGTGAACCAGAATGCCCAGGCTCTGAACACCCTGGT
GAAGCAACTTTCCAGCAACTTTGGAGCCATCTCCTCTGTGCTGAATGACATCCTGAGCAGACTGGACAAGGTGGAGGCTGAGGTCCAGATTGACAGACTGATTACAGGCAGACTCCAATCCCTCCAAACCTATGTGACCCAACAA
CTTATCAGGGCTGCTGAGATTAGGGCATCTGCCAACCTGGCTGCCACCAAGATGAGTGAGTGTGTGCTGGGACAAAGCAAGAGGGTGGACTTCTGTGGCAAGGGCTACCACCTGATGAGTTTTCCACAGTCTGCCCCTCATGGA
GTGGTGTTCCTGCATGTGACCTATGTGCCTGCCCAGGAGAAGAACTTCACCACAGCCCCTGCCATCTGCCATGATGGCAAGGCTCACTTTCCAAGGGAGGGAGTGTTTGTGAGCAATGGCACCCACTGGTTTGTGACCCAGAGG
AACTTCTATGAACCACAGATTATCACCACAGACAACACCTTTGTGTCTGGCAACTGTGATGTGGTGATTGGCATTGTGAACAACACAGTCTATGACCCACTCCAACCTGAACTGGACTCCTTCAAGGAGGAACTGGACAAATACTT
CAAGAACCACACCAGCCCTGATGTGGACCTGGGAGACATCTCTGGCATCAATGCCTCTGTGGTGAACATCCAGAAGGAGATTGACAGACTGAATGAGGTGGCTAAGAACCTGAATGAGTCCCTGATTGACCTCCAAGAACTGGG
CAAATATGAACAATACATCAAGTGGCCATGGTACATCTGGCTGGGCTTCATTGCTGGACTGATTGCCATTGTGATGGTGACCATAATGCTGTGTTGTATGACCTCCTGTTGTTCCTGTCTGAAAGGCTGTTGTTCCTGTGGCTCCT
GTTGTAAGTTTGATGAGGATGACTCTGAACCTGTGCTGAAAGGAGTGAAACTGCACTACACCGGCGCGCC

B. MFVFLVLLPLVSSQCVNLTTRTQLPPAYTNSFTRGVYYPDKVFRSSVLHSTQDLFLPFFSNVTWFHAIHVSGTNGTKRFDNPVLPFNDGVYFASTEKSNIIRGWIFGTTLDSKT
QSLLIVNNATNVVIKVCEFQFCNDPFLGVYYHKNNKSWMESEFRVYSSANNCTFEYVSQPFLMDLEGKQGNFKNLREFVFKNIDGYFKIYSKHTPINLVRDLPQGFSALEPLV
DLPIGINITRFQTLLALHRSYLTPGDSSSGWTAGAAAYYVGYLQPRTFLLKYNENGTITDAVDCALDPLSETKCTLKSFTVEKGIYQTSNFRVQPTESIVRFPNITNLCPFGEVFN
ATRFASVYAWNRKRISNCVADYSVLYNSASFSTFKCYGVSPTKLNDLCFTNVYADSFVIRGDEVRQIAPGQTGKIADYNYKLPDDFTGCVIAWNSNNLDSKVGGNYNYLYRLF
RKSNLKPFERDISTEIYQAGSTPCNGVEGFNCYFPLQSYGFQPTNGVGYQPYRVVVLSFELLHAPATVCGPKKSTNLVKNKCVNFNFNGLTGTGVLTESNKKFLPFQQFGRDI
ADTTDAVRDPQTLEILDITPCSFGGVSVITPGTNTSNQVAVLYQDVNCTEVPVAIHADQLTPTWRVYSTGSNVFQTRAGCLIGAEHVNNSYECDIPIGAGICASYQTQTNSPRR
ARSVASQSIIAYTMSLGAENSVAYSNNSIAIPTNFTISVTTEILPVSMTKTSVDCTMYICGDSTECSNLLLQYGSFCTQLNRALTGIAVEQDKNTQEVFAQVKQIYKTPPIKDFGGF
NFSQILPDPSKPSKRSFIEDLLFNKVTLADAGFIKQYGDCLGDIAARDLICAQKFNGLTVLPPLLTDEMIAQYTSALLAGTITSGWTFGAGAALQIPFAMQMAYRFNGIGVTQNVL
YENQKLIANQFNSAIGKIQDSLSSTASALGKLQDVVNQNAQALNTLVKQLSSNFGAISSVLNDILSRLDKVEAEVQIDRLITGRLQSLQTYVTQQLIRAAEIRASANLAATKMSEC
VLGQSKRVDFCGKGYHLMSFPQSAPHGVVFLHVTYVPAQEKNFTTAPAICHDGKAHFPREGVFVSNGTHWFVTQRNFYEPQIITTDNTFVSGNCDVVIGIVNNTVYDPLQPE
LDSFKEELDKYFKNHTSPDVDLGDISGINASVVNIQKEIDRLNEVAKNLNESLIDLQELGKYEQYIKWPWYIWLGFIAGLIAIVMVTIMLCCMTSCCSCLKGCCSCGSCCKFDED
DSEPVLKGVKLHYT

C. nCoV-$S_{wuhan\text{-}Hu\text{-}1}$-KpnI-F: 5’-ATATGGTACCAATGTTTGTGTTCCTGGTG-‘3
nCoV-$S_{wuhan\text{-}Hu\text{-}1}$-AscI-R: 5’-TCTGGCGCGCCGGTGTAGTGCAGTTTCACTCC-3’

Supplemental Figure S3. Nucleotide/amino acid sequences and cloning of SARS-CoV-2 Wuhan-Hu-1 spike (S) protein gene. Spike (S) protein gene of SARS-CoV-2 Wuhan-Hu-1 strain (NCBI Reference Sequence: NC_045512.2) was chemically synthesized and cloned in the plasmid pCMV3-2019-nCoV-Spike (S1+S2)-long (kindly provided by Professor Che Alex Ma at Genomics Research Center, Academia Sinica, Taiwan). Nucleotide and deduced amino acid sequences of SARS-CoV-2 S protein gene were listed (A, B), and used as the template for PCR amplification with a specific primer pair (C). Finally, the SARS-CoV-2 Wuhan-Hu-1 S protein gene was cloned into the restriction enzyme sites KpnI/AscI of pcDNA3.1/HisC-T2A-F2A-nCoV E-nCoV M.
